# Cognitive and Microbiome Impacts of Experimental *Ancylostoma ceylanicum* Hookworm Infections in Hamsters

**DOI:** 10.1038/s41598-019-44301-4

**Published:** 2019-05-27

**Authors:** Samuel C. Pan, Doyle V. Ward, Yunqiang Yin, Yan Hu, Mostafa A. Elfawal, Robert E. Clark, Raffi V. Aroian

**Affiliations:** 1 Division of Infectious Diseases, Department of Medicine, University of California, San Diego, 9500 Gilman Drive, La Jolla, CA 92093 USA; 2 Department of Microbiology and Physiological Systems, University of Massachusetts Medical School, 368 Plantation Street, Worcester, MA 01605 USA; 3 Program in Molecular Medicine, University of Massachusetts Medical School, 373 Plantation Street, Suite 219, Worcester, MA 01605 USA; 4 Veterans Affairs San Diego Healthcare System, San Diego, CA 92161 USA; 5 Department of Psychiatry, University of California, San Diego, 9500 Gilman Drive, La Jolla, CA 92093 USA; 6 Present Address: Biology Department, Worcester State University, Worcester, MA 01602 USA

**Keywords:** Spatial memory, Parasitic infection

## Abstract

Hookworms are one of the most prevalent and important parasites, infecting ~500 million people worldwide. Hookworm disease is among the leading causes of iron-deficiency anemia in the developing world and is associated with significant growth stunting and malnutrition. In humans, hookworms appear to impair memory and other forms of cognition, although definitive data are hard to come by. Here we study the impact of a human hookworm parasite, *Ancylostoma ceylanicum*, on cognition in hamsters in a controlled laboratory setting. We developed tests that measure long-term memory in hamsters. We find that hookworm-infected hamsters were fully capable of detecting a novel object. However, hookworm-infected hamsters were impaired in detecting a displaced object. Defects could be discerned at even at low levels of infection, whereas at higher levels of infection, hamsters were statistically unable to distinguish between displaced and non-displaced objects. These spatial memory deficiencies could not be attributed to defects in infected hamster mobility or to lack of interest. We also found that hookworm infection resulted in reproducible reductions in diversity and changes in specific taxanomic groups in the hamster gut microbiome. These data demonstrate that human hookworm infection in a laboratory mammal results in a specific, rapid, acute, and measurable deficit in spatial memory, and we speculate that gut alterations could play some role in these cognitive deficits. Our findings highlight the importance of hookworm elimination and suggest that finer tuned spatial memory studies be carried out in humans.

## Introduction

Hookworm disease is one of the most prevalent and important tropical diseases of humans globally and is associated with high morbidity in children and pregnant women^1–6^. In children and adolescents, infections have been linked to anemia, nutritional deficiency, growth stunting, and impaired cognition, resulting in decreased school attendance and performance, lower educational achievement, decreased wage-earning potential, and perpetuation of lower socioeconomic status and poverty^1–6^.

Although links between hookworm infection and impaired cognitive function in children have been made, the results to date have been conflicting. Some studies showed significant deficits in working memory and cognitive performance associated with hookworm infection^7–9^, while other found only minimal or no deficit at all^10,11^. Prospective, interventional studies with hookworms also showed conflicting results, with some studies showing improvement in the cognitive function scores after anthelmintic therapy or loss of parasites^12–14^ and with other studies showing no improvement or mixed results^15,16^. Given the conflicting data, it has been difficult to draw any unifying conclusions, likely due to confounding factors, such as varied region-specific parasite prevalence rate and burden, efficacy of treatment, presence of other endemic infection, nutritional status, cultural practices, socioeconomic status, and even the type and timing of cognitive tests administered^13,17,18^. Thus, development of an animal model for studying cognitive and behavioral deficits of human hookworms in a controlled environment would be very helpful.

To address these contradictory findings and difficulties in carrying out such studies in humans, we examined the impact of hookworm infections on cognition under controlled laboratory conditions. The hookworm *Ancylostoma ceylanicum* is a significant human parasite in Southeast Asia^19–21^ and, of all human intestinal nematode parasites, uniquely and readily infects and completes its life cycle in an immunocompetent laboratory rodent, the hamster^22,23^. In this study, we: (1) developed appropriate hamster behavioral assays for studying memory; (2) looked at the impact of an acute human parasitic intestinal nematode infection (overall and also segregated by infection intensity) on memory in a laboratory animal under controlled experimental conditions; and (3) looked at the impact of an experimental hookworm infection on the gut flora to explore possible mechanisms for cognitive alterations.

## Results

### Time course of infection

**Figure 1:**
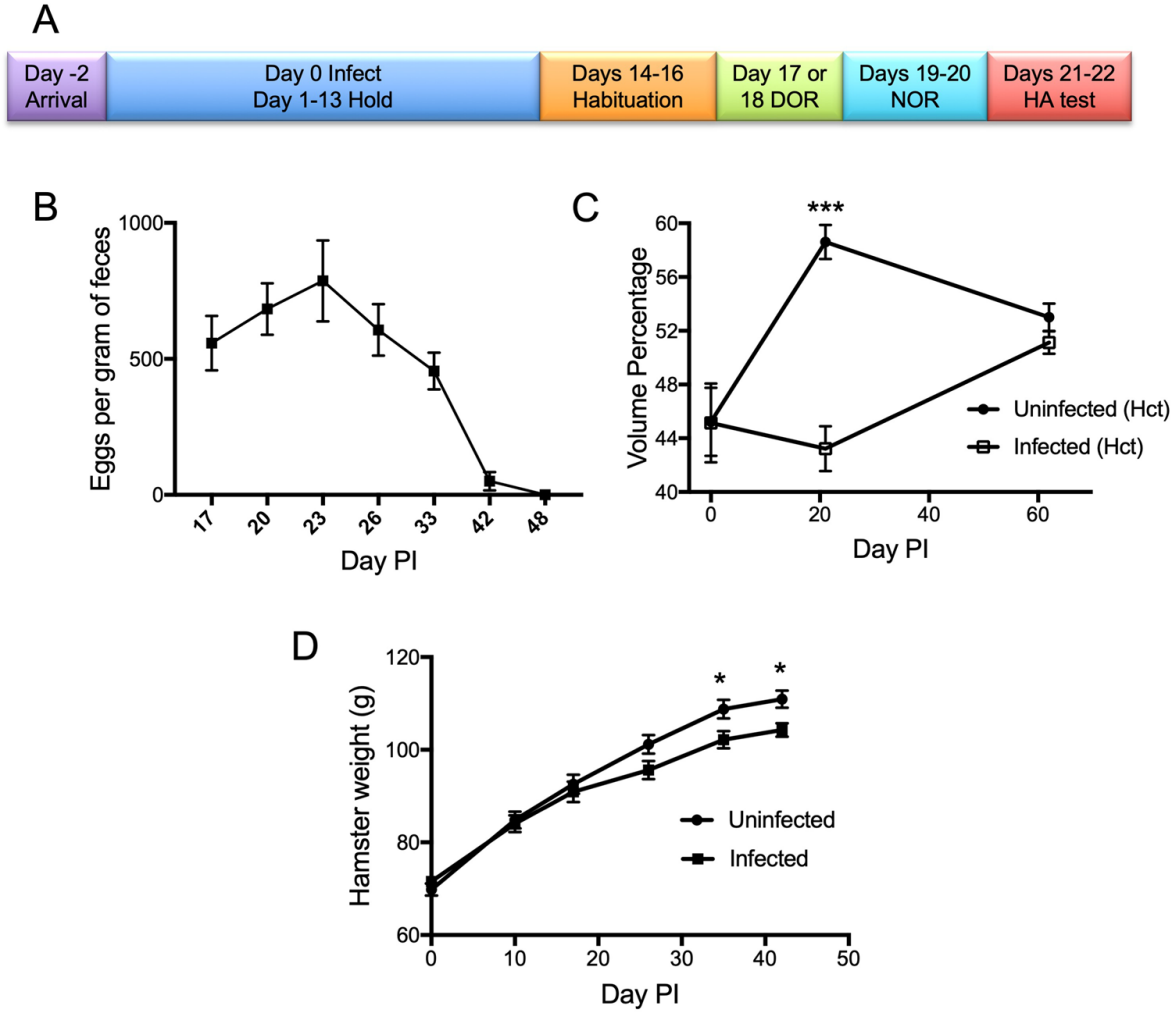
Parasitological Characteristics of Hookworm-infected Hamsters during Cognitive Studies. (**A**) Experimental Time Course. Time course of hamster infection and behavioral tasks is displayed. (**B**) Fecal egg burden over the course of infection. Fecal pellets were collected on indicated days post-infection (Day PI). Parasite egg counts are expressed as eggs per gram of feces. (**C**) Hematocrit changes over the course of infection. Blood (up to 50 µL) was collected from the superficial saphenous vein and analyzed. P values comparing uninfected to infected animals for days 0, 21, and 62 are 0.98, <0.001, and 0.23 respectively. *** indicates P < 0.001. (**D**) Weight change over the course of infection. Hamsters were weighed periodically over the time course of the experiments. P values comparing uninfected to infected animals for days 0, 10, 17, 26, 35, and 42 are 0.30, 0.77, 0.60, 0.062, 0.024, and 0.026 respectively. * indicates P < 0.05. Abbreviations: DOR–Displaced Object Recognition; NOR–Novel Object Recognition (NOR); HA–hamster activity.

Hamsters, unlike human, are capable of mounting an immune response to clear a hookworm infection without any intervention^24,25^. To establish an acute hamster infection model, the hamsters were infected with L3 stage larvae via oral gavage. Based on our prior studies, we estimated that the larvae will develop into adults beginning around day 11 or 12 post-infection (PI). As per normal infection course, egg production in this study was robust around day 17 PI (coincides with beginning of behavior tests; Fig. 1A,B) and peaked around day 23 PI, remaining robust during the entire testing phase (ended on day 22 PI; Fig. 1A,B), The infection began to significantly decline (day 33 PI; Fig. 1B) long after testing was complete. Infection was cleared by days 48–50 (Fig. 1B), in line with the known course of *A*. *ceylanicum* infection in Golden Syrian hamsters.

We also monitored the physiological impact of *A*. *ceylanicum* infection on Golden Syrian hamsters, particularly hematocrit and weight. As shown in Fig. 1C, the average hematocrit in uninfected hamsters increased up to day 21 PI and then remained stable up to day 62 PI. Infected hamsters, in contrast, were anemic and had a significant lower average hematocrit at day 21 (p =< 0.001 relative to uninfected animals), which corresponded to the peak of the infection and consumption of host red blood cells by adult hookworms. Hematocrit in the infected group improved subsequently as the infection was cleared (day 42–48 PI; Fig. 1B) and was statistically the same as hematocrit in the control group by day 62 PI.

Weight during the course of infection is shown in Fig. 1D. Infected hamsters gained less weight than uninfected hamsters over the course of infection but the difference did not reach statistical significance until day 35 PI, which was maintained at day 42 PI. We note, however, that the weights taken of infected animals were taken from a random group of 18 animals that predominantly had low burden of infection (15/18). It is therefore possible that with the entire group of animals, statistically significant difference in weight could have been reached sooner. In addition, the weights of the infected hamsters were likely aided by the fact that they were individually housed after infection. Infected animals that are housed together typically show lower weights that those individually housed. This observation suggests of a cumulative effect over time on the physical development of the host, which has been observed in children with STH infection. In those studies, physical development deficits were reversible with treatment of underlying infection and improved nutrition and hygiene. Therefore if the hamsters were monitored beyond the end of the infection course, the infected hamsters presumably should catch up with their uninfected counterparts^26^.

All behavioral assays took place between days 17–22 PI during the peak of hookworm infection to ensure maximal and consistent effects on cognitive function during all tests.

### Infected hamsters were unable to recognize spatial changes within the environment

**Figure 2:**
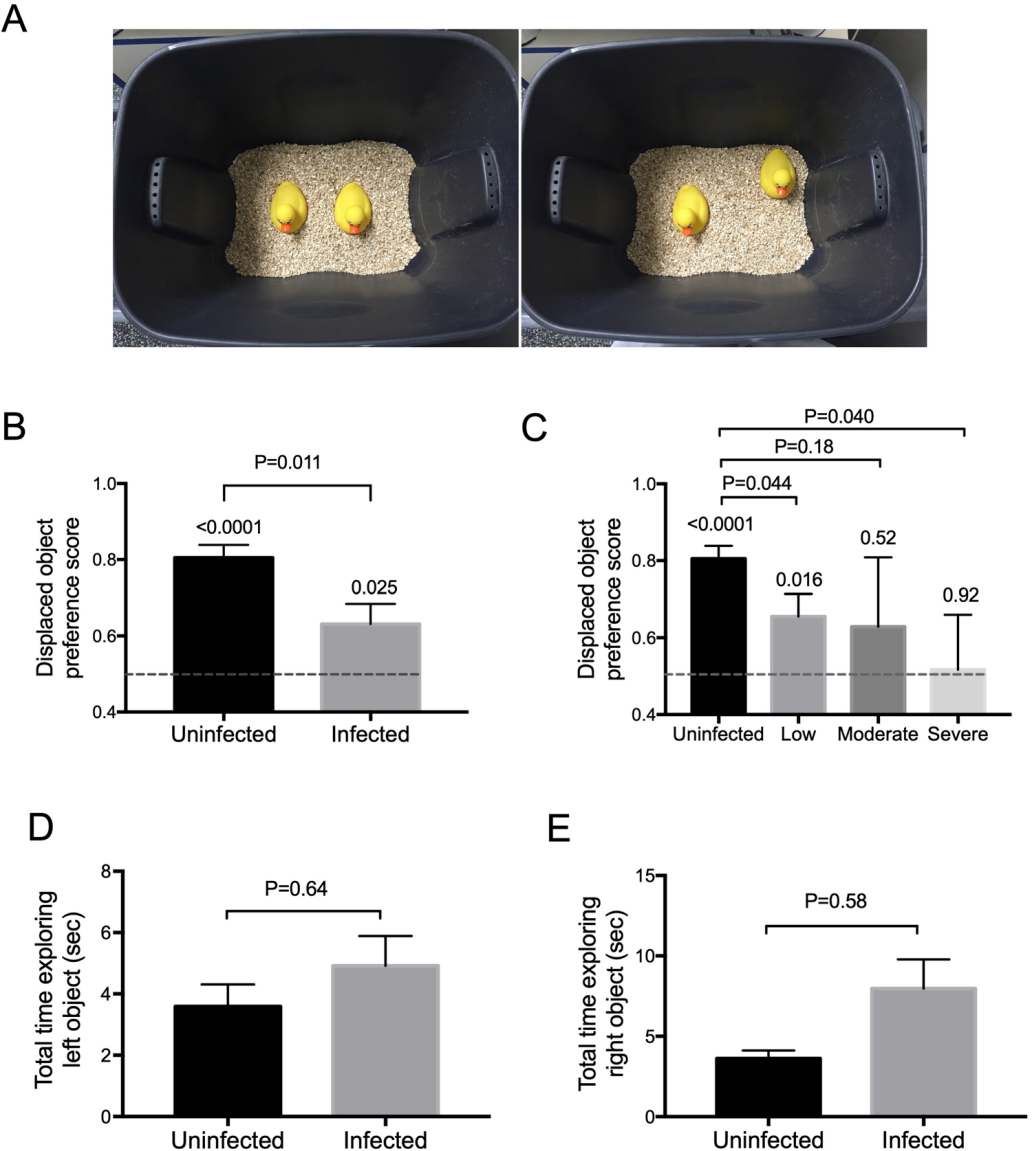
Displaced Object Recognition (DOR) task. (**A**) Experimental set-up. Hamsters were habituated to the testing environment, which constituted of a black plastic bin with identical objects, shown in the left, for three consecutive days to allow formation of spatial memory. On the day of testing, hamsters were familiarized to the testing environment again. After a period of rest, one of the two identical objects was displaced to a corner that was randomly determined, shown in the right. The hamster was then assessed for their ability to detect the change in the environment, which was recorded for playback and scoring at a later time. (**B**) Infected hamsters performed worse than uninfected hamsters in the DOR task. The dash line here and in (**C**) represents displaced object preference score of 0.5 or equal preference toward displaced and non-displaced objects. The P value above each individual bar represents a comparison of the performance of each group relative to no preference (displaced object preference score of 0.5). Infected hamsters showed a weaker preference towards the displaced object and performed significantly different when compared to the uninfected hamsters (p = 0.011). (**C**) Severity of performance deficit broken down by infection burden. Hamsters were divided into 3 infection burden groups: low, moderate, and severe. The P value above each individual bar represents a comparison of the performance of each group relative to no preference (displaced object preference score of 0.5). Both the moderate and severely infected hamsters showed no preference toward the displaced object. The low infected group showed a weaker preference towards the displaced object relative to uninfected animals. Infected animals in the low and severe infected groups performed significantly different when compared to the uninfected hamsters (p = 0.044 and 0.040 respectively). (**D**) Both uninfected and infected hamsters spent similar amount of time exploring the objects. The amount of time (in seconds) each hamster spent exploring each object during the familiarization phase was calculated during the first 10 minutes of the task. There was no significant difference in time of exploration between infected and uninfected animals for either object.

To study the impact of hookworms on memory, we developed several hamster behavioral tasks that were designed to measure memory, which were adapted from the rat model system. The first area of cognition we assessed was the animal’s ability to form a spatial memory and detect a change in object placement within the environment using the Displaced Object Recognition (DOR) task. The hamster was first habituated to the test environment with two identical objects for 15 minutes daily for 3 consecutive days (Fig. 2A). The repetition allowed the hamsters to form memory of a spatial map of the environment. On the day of testing, the hamster was familiarized to the same environment to reinforce the memory and after a 3-hour rest period, the hamster was placed back into the environment, but with one of the objects shifted to the corner. If the hamster has successfully formed spatial memory, then it would recognize the shift in the object’s position as a change in the environment and therefore preferentially spend more time exploring this change.

As shown in Fig. 2B, the uninfected hamsters demonstrated expected preference for the displaced object, recognizing its novelty in the environment (P < 0.0001 compared to no preference, a displaced object preference score of 0.5). However, while infected hamsters were also able to perform the task (P = 0.025 compared to a displaced object preference score of 0.5), they showed significantly less preference compared to uninfected hamsters (p = 0.011).

When broken down further by intensity of infection, the differences are even more striking, showing a decrease in performance with increased severity of infection. Analysis of variance comparing the displaced object preference score of uninfected animals to displaced object preference scores in each of the infected groups reveals that both low and high intensity hookworm infection result in reduced displaced object preference relative to uninfected controls (lack of significant difference between controls and moderately infected animals seems to reflect the wide standard deviation, which likely would improve with higher n). Moreover, while the uninfected and low infected group performed the task and show a preference towards the displaced object (respectively, P < 0.0001 and P = 0.016 relative to a displaced object preference score of 0.5), neither the moderately infected nor the severely infected groups showed a preference towards the displaced object (respectively, P = 0.52 and 0.92 relative to a displaced object preference score of 0.5) and thus failed to exhibit spatial memory.

Since the ability to perform the task depends on a hamster’s motivation to explore and perceive objects in order to form a spatial map of the testing environment, one can argue that hookworm infection might affect the animal’s ability or willingness to explore the environment or perceive the object during habituation. Thus, for example, the animal may not have had sufficient exposure to allow for spatial memory formation nor recognition of displaced object. Furthermore, since hookworms can cause lethargy, we wanted to determine if strong differences in movement might account for differences in uninfected vs. infected animals to perform the task.

To address this issue, we evaluated the amount of time each hamster spent exploring each object in the first 10 minutes of the corresponding familiarization period (non-displaced object training). As shown in Fig. 2D, there was no difference in the amount of exploration time between uninfected and infected hamsters on either object during the familiarization phase (P = 0.64 and P = 0.58 for left and right objects respectively). These data suggest that neither lack of interest, lack of mobility, nor inability to detect an object was responsible for the differences between uninfected and infected hamsters in the DOR assay.

To further confirm that lack of activity or interest was not responsible for the differences seen in the actual task, we also looked at the percent of animals that met the inclusion criteria for the DOR task, *i*.*e*., explored both objects combined at least a total of 10 seconds (see Material and Methods). For uninfected animals, 35 out of 64 met the inclusion criterion whereas 29 out of 51 infected animals met the inclusion criterion. There was no significant difference between uninfected and infected groups for the percentage of animals that successfully met the inclusion criterion for (P = 0.86 using Fisher’s exact test). This finding again supports the conclusion that differences seen in displaced object preference scores between groups was neither due to a lack of interest nor a lack of mobility in the infected group. We conclude that hookworm infection causes a deficit in the hamster’s ability to properly form spatial memory, namely the inability to recognize a shift in position as a change in the environment.

### Hookworm-infected hamsters were able to recognize novel object

**Figure 3:**
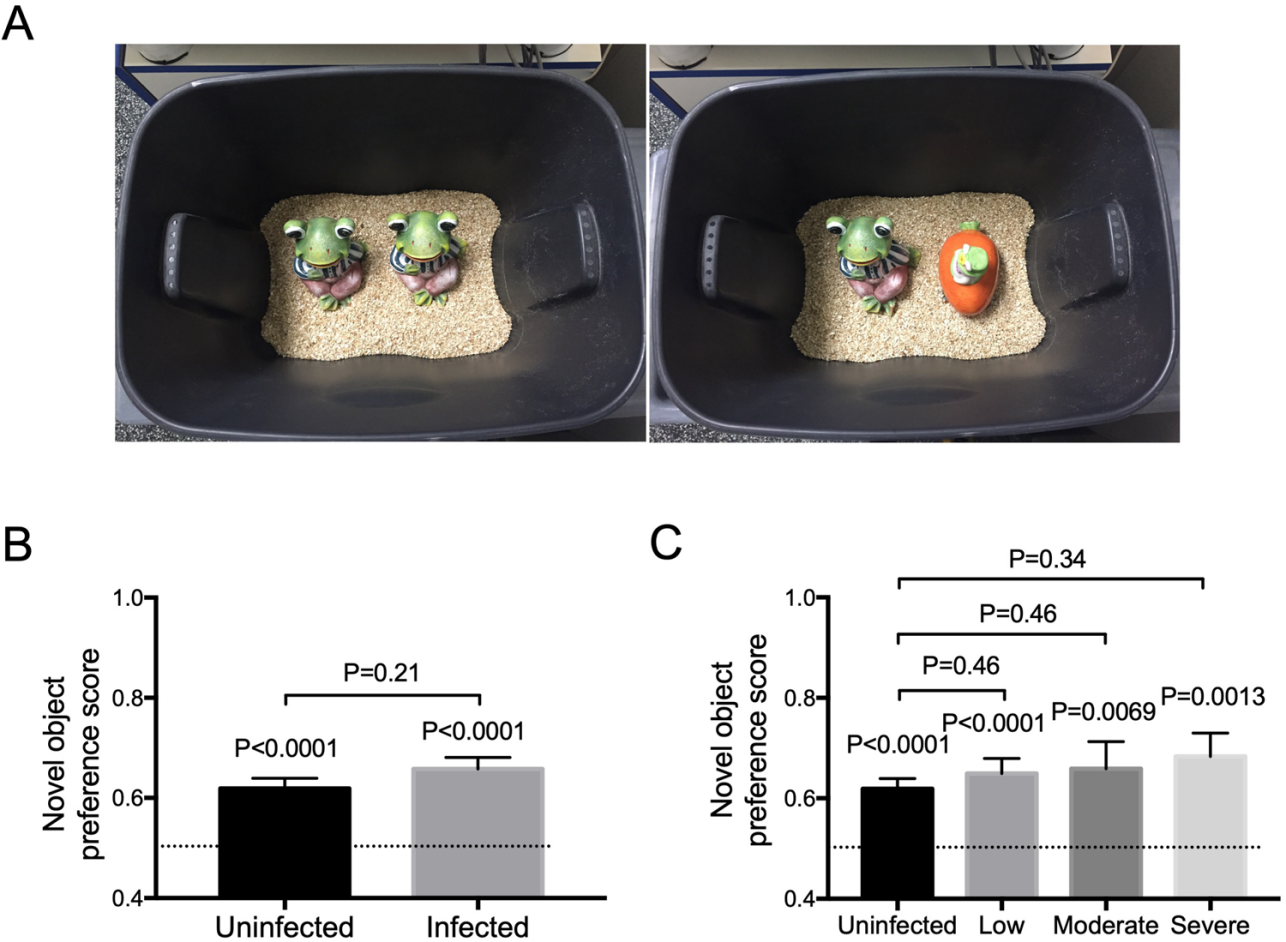
Novel Object Recognition (NOR) task. (**A**) Experimental set-up. Using the same testing environment to which the hamsters were previously habituated, the hamsters were familiarized with two identical objects for 15 minutes on the day of testing. After the resting period, one of the objects was replaced with a novel object, as shown in the right. The hamsters were assessed for their ability to preferentially explore the novel object over the familiar object. The experiment was repeated the next day with two completely different objects. (**B**) Infected hamsters performed the same as uninfected hamsters in the NOR task. The dash line here and in (**C**) represents novel object preference score of 0.5 or equal preference toward the novel and familiar objects. The P value above each individual bar represents a comparison of the performance of each group relative to no preference (novel object preference score of 0.5). Infected hamsters showed the same preference towards the novel object and performed the same when compared to the uninfected hamsters (p = 0.21). (**C**) Performance broken down by infection burden. Hamsters were divided into 3 infection burden groups: low, moderate, and severe. The P value above each individual bar represents a comparison of the performance of each group relative to no preference (displaced object preference score of 0.5). All hamsters regardless of infection status or intensity showed significant preference toward the displaced object. Infected animals regardless of intensity of infection behaved similarly to uninfected controls.

Given the DOR task results, we wanted to see if a hookworm infection would also have an effect on object recognition, a nonspatial task. This would also help determine if behavioral defects in infected hosts were general or specific. Using a similar testing environment but with a different object set up, we tested the hamsters in the Novel Object Recognition (NOR) task. In this task, the hamsters were familiarized to 2 identical objects (Fig. 3A) on the day of testing. After a 3-hour resting period to allow for object memory formation, the hamsters were returned to the environment with one of the now familiar objects replaced with a completely novel object (*e*.*g*., Fig. 3A). The side of novelty for each hamster was randomized to minimize any potential side bias. Both the familiar and novel objects were selected for their similar sizes and color to avoid potential bias. The assay was repeated on the following day with a completely different set of novel objects (not shown). The data from both days were combined and used in the analyses.

We predicted that, as with rats, uninfected hamsters would recognize novel object as different from its prior experience and preferentially spent more time exploring it. Indeed, this was the case with uninfected hamsters showing a novel object preference score of 0.62 (Fig. 3B), which was statistically different (P < 0.0001) from no preference (novel object preference score of 0.5).

Surprisingly, infected hamsters behaved similarly to uninfected hamsters in the NOR test. Pooling all infected animals independent of infection intensity, there was no difference in novel object preference scores of uninfected versus infected hosts (P = 0.21). As with uninfected animals, infected animals showed a preference for the novel object (novel object preference score of 0.66; Fig. 3B) that was statistically different (P < 0.0001) from no preference (novel object preference score of 0.5).

When broken down by infection intensity, the NOR results did not change (Fig. 3C). There was no statistical difference in novel object preference scores between uninfected hosts or infected hosts in any infection-intensity category, and all novel object preference scores were significantly different than 0.5 (i.e., all animals showed a preference for the novel object irrespective of infection status or intensity). As with the DOR task, there was no significant difference between uninfected (118/130) and infected (97/102) groups for the percentage of animals that successfully met the inclusion criterion for the NOR task (P = 0.31 using Fisher’s exact test). All in all, we conclude that uninfected and infected hamsters were able to perform the task and that hookworm infection did not hamper the hamster’s ability to form object memory (Fig. 3B,C).

### Hookworm infection did not affect normal activity level

It has been reported that children with hookworm infection tend to be more lethargic and uninterested, which can sometimes be seen with hookworm-infected hamsters as well. Our data looking at exploration during the DOR habituation phase and the percent of animals that met the inclusion criterion of >10 seconds of exploration for both uninfected and infected animals indicate that lethargy and lack of interest do not account for our results. Consistent with this, we also find that infected hamsters were completely capable of performing the NOR task as well as uninfected animals. If they had overall movement defects or lethargy, we would have predicted that would have failed this test as they did DOR. However, the NOR task was performed normally.

**Figure 4:**
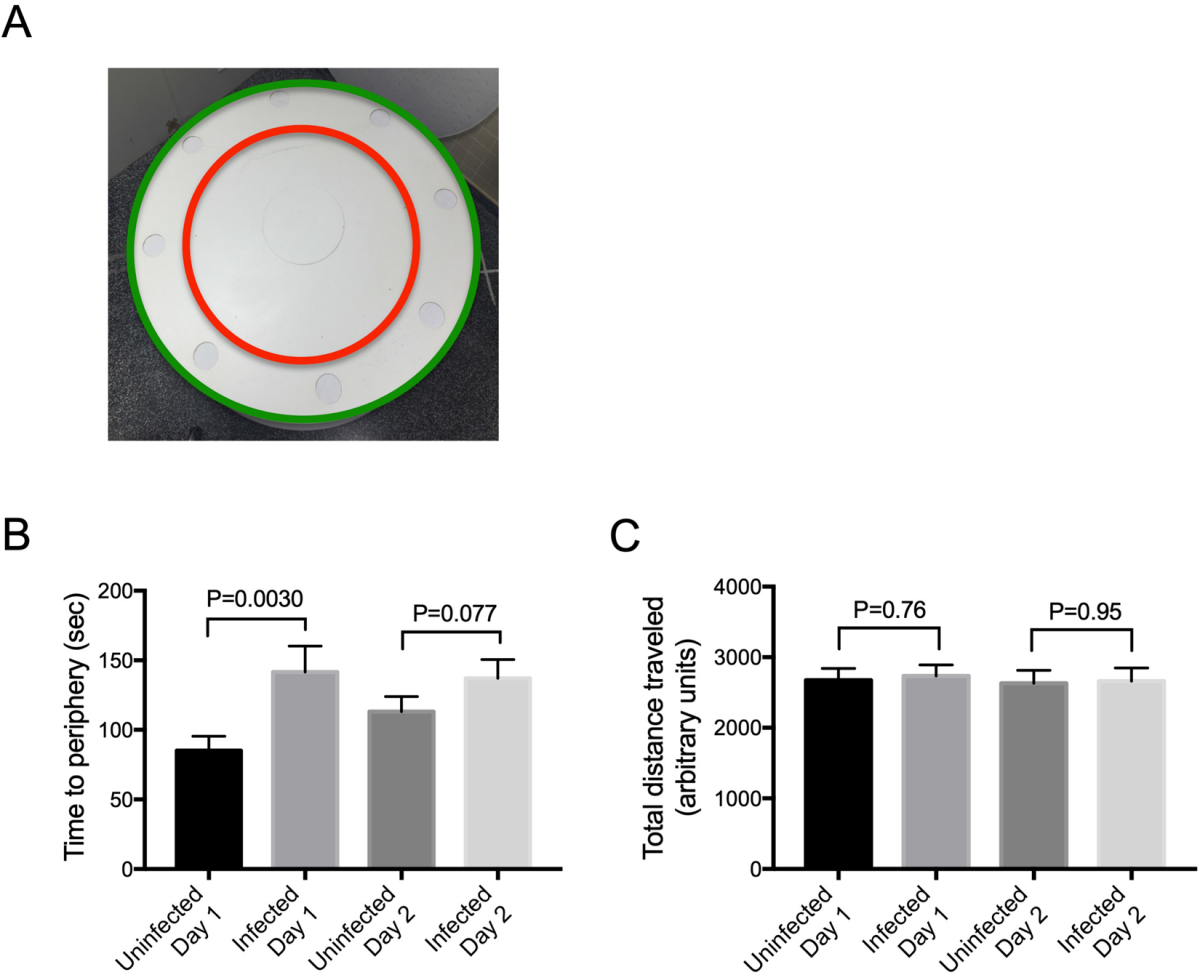
Activity Task. (**A**) Experimental set-up for Activity Task. Hamsters were placed in the center of a round table within a closed room. The hamsters were allowed to explore the table for 10 minutes and the entire task was recorded for later scoring. The task was repeated on a second day. The time each hamster took to go from the center to the border of Central (**C**) and Peripheral (P) zones and the total distance traveled was calculated from recordings. The green and red circles were not present during hamster testing but were added by computer during image analyses. (**B**) Time to get to the Peripheral zone. The amount of time in seconds it took for a hamster to go from starting point (center of the table) to the boundary between Central and Peripheral zones was determined and plotted for each day. On day 1, but not day 2, infected animals took longer to reach the periphery (P = 0.0030). (**C**) Uninfected and infected hamsters traveled similar distance in the Activity task. Total distance (arbitrary units) traveled within both Central and Peripheral zones were recorded and calculated. There was no difference in distance traveled between infected and uninfected hamsters on either day.

To address this issue independently, after the NOR task, we set up an Activity Task for the hamsters that, at least initially was meant to be used as a habituation to attention (HTA) task (see Materials and Methods). A hamster was placed on the center of an open table (Fig. 4A) within a closed room and allowed to explore the table or the “environment” for 10 minutes every day for 2 consecutive days. The movement of the hamster over the area of the table was tracked and recorded using an overhead camera for subsequent analyses. The amount of time each hamster took to move from the center (Central Zone, red inner circle) to the edge of the table (Peripheral Zone, green outer ring) was determined. The total distance traveled by each hamster was also determined.

Although uninfected hamsters did not behave as predicted based on rat studies (see Materials and Methods), the data were nonetheless useful to assay hamster activity. When measuring the amount of time it took the hamsters to find the Peripheral Zone, relative to uninfected hamsters, infected hamsters took slightly longer on day 1 (P = 0.0030) but not on day 2 (P = 0.077; Fig. 4B). However, on both days, there was no difference in the total distance traveled between uninfected or infected hosts on either day 1 or day 2 in the allotted period of time (Fig. 4C). Thus, in agreement with our analysis of interest and activity in the DOR assay, the activity test indicates there are no obvious overall defects in infected hamster attention and activity that would account for the deficits seen in the DOR task.

### Hookworm infection impacted the gut flora

In recent years, there is increasing evidence to suggest a link between the gut, the gut intestinal flora, and behavior/neurocognitive function (see Discussion), raising the possibility that some of the behavior deficits could be due to changes in gut physiology and/or the gut flora. We decided to investigate the impact of hookworm infections on the gut microbiome to determine if *A*. *ceylanicum* hookworms were having a significant impact on gut physiology. Two studies of the impact of hookworms on the microbiome have been carried out in celiac patients^27,28^, but no studies on the impact of human hookworms on the microbiome in this experimental hamster-hookworm system or on non-diseased hosts. To ascertain whether or not our experimental hookworm infection resulted in changes in the microbiome, we compared the microbiome of infected and non-infected animals 32–33 days post-inoculation in two independent experiments.

**Figure 5:**
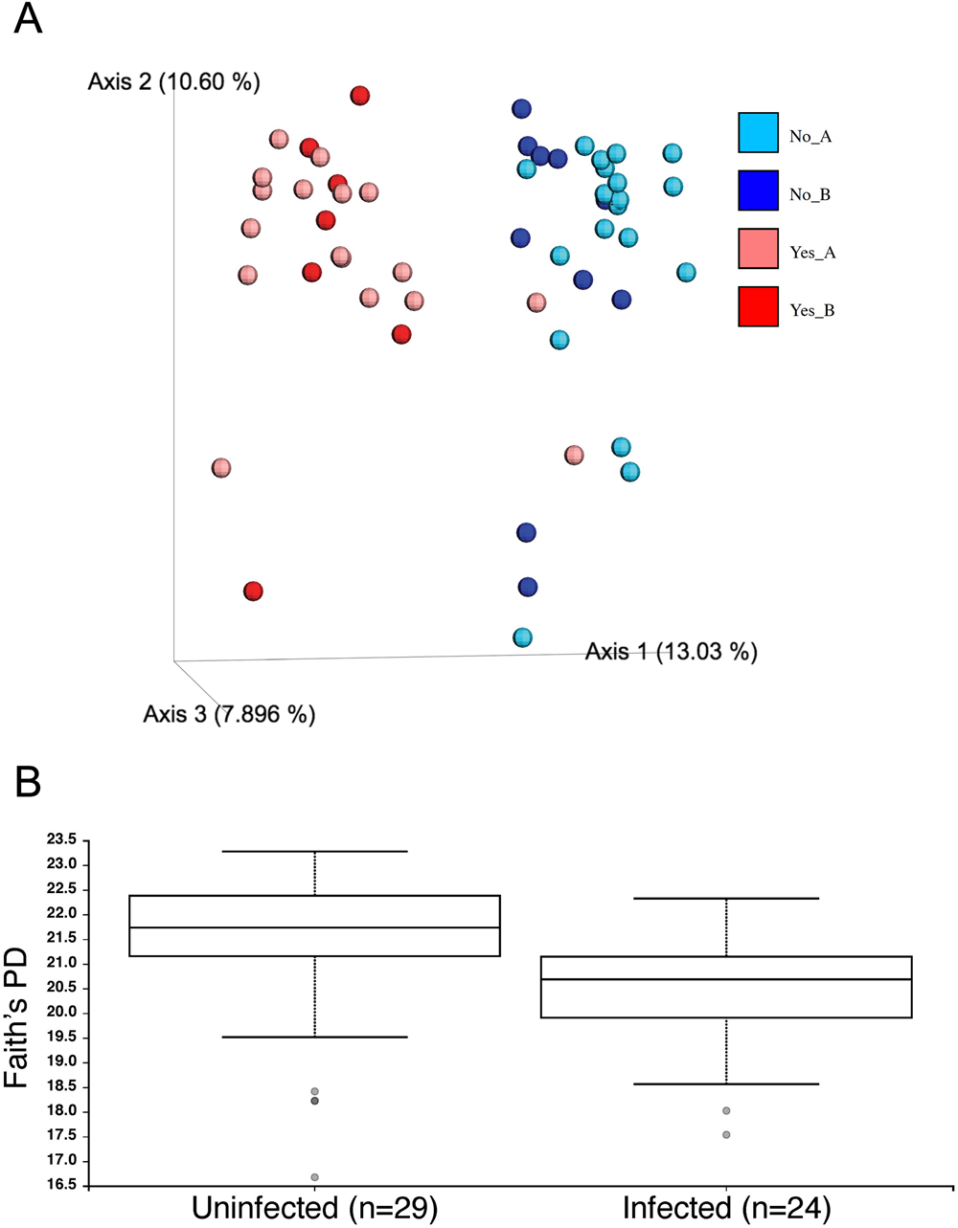
Microbiome analyses of hookworm-infected hamsters. (**A**) Beta-diversity between hookworm-infected and uninfected hamsters. Principal Coordinate analysis of beta-diversity between samples is presented as calculated in QIIME2:2018.4. The unweighted unifrac metric was used to calculate distances between samples. The percent of total variation explained by each PCoA axis is given in parentheses. Red tones = infected; blue tones = uninfected. Experiments A and B are shown separately for each. Each dot represents an individual sample. (**B**) Alpha-diversity between hookworm-infected and uninfected hamsters. Alpha diversity analysis using Faith’s Phylogenetic Diversity (PD) as calculated in QIIME2:2018.4. Boxes indicate the 25th and 75th percentiles (first and third quartiles) for uninfected (left) and infected (right) hamsters. The horizontal line within the box denotes the median. Whiskers show the range of the data excluding outliers (which are shown as gray dots) that fell more than 1.5 times the interquartile range.

The composition of gut flora was significantly different between infected and uninfected animals (unweighted unifrac; PERMANOVA, P < 0.001) (Fig. 5A). Furthermore, alpha diversity was significantly lower in infected animals (Faith’s PD, p = 0.002) (Fig. 5B). At the family and genus level, a limited number of taxa differentially enriched both in infected and in uninfected hamsters were detected (Table 1).

**Table 1:** Differentially enriched taxa in infected and uninfected hamsters.

Infected	Eubacteriaceae
	Sutterellaceae
	Eubacteriaceae:*Eubacterium*
	Lachnospiraceae:*Coprococcus*
	Lachnospiraceae:*Clostridium*_XlVb
	Porphyromonadaceae:*Parabacteroides*
	Porphyromonadaceae:*Barnesiella*
	Sutterellaceae:*Parasutterella*
Uninfected	Bifidobacteriaceae
	Bifidobacteriaceae:*Bifidobacterium*
	Erysipelotrichaceae:unclassified
	Lachnospiraceae:*Roseburia*
	Lachnospiraceae:*Acetatifactor*

ANCOM was used to text group significance at both the family and genus levels. Of the 56 genus level and 32 family level features considered significance is reported for W > 40 and W > 27, respectively.

## Discussion

Here we studied the impact of an acute experimental human hookworm infection in hamsters on cognition and memory in a controlled laboratory setting. The ability of the hamsters to recognize a new object (Novel Object Recognition or NOR) was unaffected by hookworm infection regardless of the infection intensity of the hamsters.

However, in a measure of spatial memory, hookworm-infected hamsters were compromised in their ability to detect object displacement (Displaced Object Recognition or DOR) even at low levels of infestation. At higher levels of infestation, the animals failed the task–they had no statistical preference for either the displaced and non-displaced objects.

This failure of the infected hamsters to perform normally on the DOR task could not be attributed to lethargy or lack of general interest. First, the infected hamsters performed normally on the Novel Object Recognition (NOR) task. Second, in our hamster activity (HA) task both groups of hamsters were indistinguishable. Third, for both the NOR task and the DOR task, the same portions of infected and uninfected animals identically met the inclusion criterion for minimal exploration time. Fourth, during the familiarization phase for the DOR task, both infected and uninfected groups equally explored each of the objects. We therefore conclude that hookworm infection does not affect all memory functions adversely but, more specifically spatial memory. We did not note any physical changes at the level of gross morphology of the brain when we examined the brain tissue from a few hamsters, both uninfected and infected (data not shown).

Our results showing that an acute hookworm infection caused defect in spatial memory are remarkably similar to other findings with intestinal nematode infections in the laboratory. Infections with both *Heligmosomoides polygyrus* in mice and *Nippostrongylus brasiliensis* in rats also resulted in defects in spatial learning and spatial reference memory^29,30^, although such an effect was not seen with *Strongyloides ratti* infections^31^.

It is stunning that three different intestinal nematode infections in three different rodents all led to spatial memory defects, suggesting that spatial memory defects might be a conserved feature of these infections. It is therefore important to extend these findings to studies in humans, and, given recent work on experimental hookworm infections in humans to treat autoimmune diseases^32^, it might be possible to conduct such human studies under more controlled settings. Although these autoimmune studies are being done with low levels of hookworm infection, our findings suggest that even light hookworm-infected animals have measurable but subtle cognitive changes.

This study, in addition to its striking findings, provides useful information for future cognitive studies in hamsters and in the hamster-hookworm system. It is worthwhile to note that, although we successfully worked out conditions to test NOR, DOR, and HTA tasks with hamsters, we found that hamsters were not amenable to evaluation with two other benchmark tests of cognitive function commonly used in rat and mouse studies. Namely, the hamster’s swimming ability was too weak to be reliably tested on the water maze task, a benchmark test of spatial memory^33^, and following context fear conditioning, we were unable to measure a fear memory as traditionally indexed by a freezing response^34^. However, both the DOR and NOR tests work well in hamsters and the HTA test can be adapted to a generalized motor/motility test (HA).

Determining the exact mechanism would have been challenging to study given the effort in establishing our novel behavioral assays in hamsters, our lack of knowledge ahead of time of what the results would be, and the number of animals involved (n = 115). Nonetheless, the phenotype of the memory impairment, where spatial memory is impaired while object recognition memory is spared, is highly suggestive that hippocampal dysfunction is responsible for the findings presented here. First, spatial tasks are exquisitely sensitive to hippocampal damage or disruption and the evidence for this is one of the most consistent findings in the field of memory research (for extensive review see^35^). Second, while hippocampal damage impairs both spatial and object memory, it takes more hippocampal disruption to impair object memory compared to spatial memory^36^, a phenotype not seen with damage to extra hippocampal structures. Accordingly, our findings have identified the hippocampus as a prime target brain structure for further research in this area.

Our results raise the question of how an acute intestinal hookworm infection can cause defects in memory. Intestinal nematodes infect the gut, and it is known that the gut can have profound impacts on brain function, cognition, memory, and behavior^37^. The gut flora can also participate in modulating all these brain functions, *e*.*g*., by altering the production of neuropeptides, neurohormones, and neuroactive substances that cross the blood-brain barrier^37^. Furthermore, there is mounting evidence that the microbiome can specifically impact spatial memory and learning^38–44^. We therefore investigated if hookworm infections in hamsters caused significant changes in the gut vis-a-vis the gut flora. Hookworm infection in hamsters led to decreased microbial diversity and changes in microbial taxa at the family and genus level. Our results confirm that the hookworms are having a significant impact on the hamster gut and microbiome, as ascertained by changes in the gut flora. These changes provide one possible mechanism that contribute to spatial memory defects. To our knowledge, this is the first study of the impact of human hookworms on the microbiota of a healthy host.

Our results support the importance and urgency of global deworming efforts and eliminating hookworms in order to alleviate poverty and in “saving brains” (improving cognitive function) for impoverished children. The findings presented here indicate that hookworm infection affects cognitive function in a specific manner and that either (1) spatial memory formation is affected to a greater extent than object memory or (2) there is an inability to recognize subtle changes within the task but not obvious changes. Our studies also point to an emerging trend in association between intestinal nematode infection and spatial memory defects. These results may also explain, in part, the inconsistency in the specific deficits that have been observed over the years with human behavioral studies and suggest that more fine-tuned human behavioral studies on spatial memory be carried out in the field. Cognitive impacts of hookworms demand additional research in order to help us understand how infection affects cognitive development and function.

## Methods

### Animals

Male Golden Syrian hamsters (3–4 weeks old) were purchased from Harlan Laboratories. The animals were allowed to rest and acclimate to the animal facility 48 hours prior to inoculation. All animal experiments were carried out under protocols approved by the University of California, San Diego and University Massachusetts Medical School Institutional Animal Care and Use Committees (IACUC protocols S09067 and A-2483–14 respectively). All housing and care of laboratory animals used in this study conform to the NIH Guide for the Care and Use of Laboratory Animals in Research and all requirements and all regulations issued by the USDA, including regulations implementing the Animal Welfare Act (P.L. 89–544) as amended. Male hamsters were used for all studies because female hamsters are >5-fold less susceptible to *A*. *ceylanicum* infection (manuscript in preparation). Using females would require vastly larger numbers of animals to achieve adequate infection intensity, prevalence, and statistical significance and would have made the study impractical.

For the behavioral studies, after initial testing trials to establish hamster behavioral protocols, we decided on 16 animals/group/experiment as a manageable number to test with a target of five experimental groups total. As the study progressed, we increased the number of groups to seven in order to ensure that we had enough infected animals in each of the categories (severe, moderate, low infectious burden). The total number of animals in the behavioral study was 115 (64 uninfected; 51 infected). The total number of animals in the combined microbiome studies was 20 uninfected and 16 infected.

### Hookworm infection

The *A*. *ceylanicum* life cycle was maintained as previously described using 3–4 week old Syrian hamsters^22,45^. Hookworm infection for experiments was done using gavage of 150 infectious *A*. *ceylanicum* larvae at L3 stage in accordance with a previously established protocol^45^. Each control animal was gavaged with solution minus larvae. Animals were individually housed after infection.

Fecal pellets then were collected every 4–5 days, starting on day 17 post-infection (PI), to assess the establishment and intensity of infection, using the McMaster method^46^. Low infectious burden was categorized as 1000 EPG or less, moderate infection was categorized as 1000–1750 EPG, and severe infection was categorized as more than 1750 EPG. Fecal egg counts (FECs) were taken for all animals on day 17 PI and then variably from between day 20- day 33 PI (exact day varied from group to group but was always the same within each group). The highest FEC seen was used to establish infectious burden status based on the above criteria. In the low, moderate, and severe groups, we ended up with 29, 14, and 8 animals respectively. Most animals were sacrificed on day 28 PI at the conclusion of all testing, except for a few that were maintained longer to continue monitoring the course of infection. For Fig. 1B, the number of animals used for FECs on each day are: n = 51 (day 17 PI); n = 18 (day 20 PI); n = 19 (day 23 PI); n = 30 (day 27 PI); n = 18 (day 33 PI); n = 6 (day 42 PI); n = 6 (day 48 PI).

The hamsters were monitored for their overall health through visual inspection. To evaluate the effect of the hookworm infection, weight measurements were obtained on day 0, 10, 17, 26, 35, and 42 PI on a subset of the hamsters (Uninfected N = 14, Infected N = 18, except for day 42 where uninfected N = 10 and infected N = 6). Similarly, blood samples were obtained from a small group of hamsters (Uninfected N = 10, Infected N = 6) to evaluate the anemia effect due to hookworm infection. Blood samples for hematocrit were obtained on days 0, 21, and 62 PI. Blood samples were obtained with the help of UCSD Animal Care Program (ACP) personnel. Each hamster was immobilized and the saphenous vein on the inner thigh was identified and punctured with a sterile hypodermic needle. Approximately 50 µL of blood was collected into EDTA coated mini capillary microtainers (Ram Scientific #076011, Yonkers, NY). The samples were processed by the UCSD ACP diagnostic lab according to standard protocol. In the day 21 PI samples, there were technical issues with two control and one infected (Uninfected N = 9, Infected N = 5 for that day).

### Behavioral methods

#### Habituation phase

Each hamster was habituated to the testing box and objects, starting on day 14 or 15 post-infection (PI) for three consecutive days. Each day of habituation was started by holding each hamster for 15 minutes by the same tester who would later administer the behavioral tasks. This was done so that the hamsters would be comfortable to being handled by the human tester, the hamster was then placed in the testing box, which consisted of a black plastic storage container without the lid (48.9 cm × 35.6 cm × 34.3 cm). A 40-watt incandescent light bulb provided illumination for the room. Two identical yellow rubber objects (~10 cm × 7 cm) were placed side by side in each container in the same orientation. Each hamster was placed into the container for 15 minutes every day for 3 consecutive days. An overview schematic of the timeline of behavioral tests for the hamsters is shown in Fig. 1A.

#### Displaced object recognition (DOR) task

The DOR task tests the hamster’s ability to form a spatial map of its environment. For the task, two identical objects were placed in an environment and the test hamster was allowed 15 minutes to become familiarized with this environment. The hamster was then returned to its cage to rest for 3 hours, to allow for formation and consolidation of spatial memory. After the rest period, the hamster was returned to the same test environment with the same two objects, however now with one object displaced to one of the corners closest to its original position. If the hamster was able to successfully form spatial memory of the environment with positions of those object, it should notice the displaced object and considered it as novel, thus preferentially explored it over the non-displaced object.

On the day of testing immediately following the final day of habituation (either day 17 or 18 PI), each hamster was again familiarized to the testing environment with the identical objects as during the habituation phase. Following this familiarization phase, the hamster was returned to its cage for a 3-hour delay interval. The hamster was then placed back into the same container with the same objects, but now with one of the objects displaced to one of the corners closest to its original position. The side and corner of displacement were counterbalanced. The hamsters were allowed to explore the environment and object for 15 minutes. Both familiarization and testing phases were recorded by an overhead camera for scoring and analysis. Object exploration was scored from video recordings by an experimenter who was blind to the group membership of the animals during testing and during off-line data analysis. Object exploration was scored when the animal’s nose was within 1 cm of the object^47^. Object exploration was not scored when the animal used the object to rear upward. Scoring was continued during video playback until a total of 30 seconds of exploration of both objects was reached for each hamster. The preference for the displaced object was expressed as the displaced object preference score which is the percent time that the hamster spent exploring the displaced object compared to the non-displaced object (displaced object preference score = time exploring displaced object/time exploring both objects). Hamsters that did not reach a minimum of >10 seconds total exploration during the 15-minute recording were excluded from analyses. Therefore, all hamsters analyzed accumulated a total of 10 to 30 seconds of exploration time. To demonstrate that the hamsters were active and engaged in object exploration, we examined the total amount of time hamsters explored each of the objects during the familiarization phase for the entire 15 minutes. Because some of these animals did not go pass the inclusion criterion of >10 seconds exploration during the testing phase, there are more animals Fig. 2D than in Fig. 2B,C.

#### Novel object recognition task (NOR)

The NOR task tests an animal’s ability to recognize an object^33^. For the task, two identical objects were presented to the animal on the day of testing, either on day 18 or 19 PI. After a 15-minute familiarization phase, the hamster was returned to its cage for a 3-hour rest interval, which allowed time for the hamster to form object memory. After the 3-hour rest period, the hamster was placed back into the same container but with one of the objects replaced by a new object. The hamster was allowed to explore for 15 minutes, as in DOR task. The NOR task was administered and scored in the same way as the DOR task. Both familiarization and testing phases were recorded by an overhead camera for subsequent scoring and analysis. Object exploration was scored when the animal’s nose was within 1 cm of the object^47^. Object exploration was not scored when the animal used the object to rear upward. The preference for the novel object was expressed as the novel object preference score which is the percent of time the hamster spent exploring the novel object compared to the familiar object (novel object preference score = time exploring novel object/time exploring both objects). NOR testing was repeated with a different set of objects the following day (day 19 or day 20 PI). Hamsters that did not reach a minimum of >10 seconds total exploration during the 15-minute recording were excluded from analyses. Therefore, all hamsters analyzed accumulated a total of 10 to 30 seconds of exploration time. The results from both days for each animal was used in the analyses.

#### Activity test

This test is based upon the habituation to attention (HTA) test originally developed for rat behavioral studies. The goal of the test is to evaluate the subject’s ability to form memory of its environment. The animal is placed in an environment and allowed to freely explore for 10 minutes. The process is repeated on day 2. The exploration on both days are recorded by an overhead camera. The amount of time it takes for the animal to reach the Peripheral Zone from the starting point and the total distance traveled each day are analyzed and compared. If the subject is able to perform the task as expected, *i*.*e*., forms memory of the testing environment, then the animal should spend more time getting to the Peripheral Zone and travel a shorter total distance on day 2 compared to day 1, as it has become familiar to the testing environment and thus is less interested in further exploration of the environment. However, we found that the hamsters did not performed the task as expected— there was no increase in time to Peripheral Zone and no decrease in total distance traveled in uninfected control animals on the second day. Thus the results from the task were interpreted in terms of overall hamster activity, comparing time to periphery and the total distance traveled in uninfected and infected animals on both days, labeling it “Hamster Activity” (HA) test.

For the HA test, the testing room was set up with a round table with a diameter of 177 cm. The table was painted plain white to remove any potential visual cue; the red and green circles shown in Fig. 4A were added by computer during image processing for automated scoring of the task. The hamster was placed in the center of the table and allowed to explore the table for 10 minutes on 2 consecutive days. An overhead camera was used to track and record the movement of each hamster. After the test was complete, a computer program was used to divide the surface of the table was into a fixed outer ring (Peripheral Zone) and an inner circle (Central Zone). For the HA test, a 10-minute testing period for each hamster was recorded by an overhead camera and converted into digital data, which was analyzed by commercial software (Smart Tracking, San Diego Instruments) to extract the relevant data. The time for each hamster to reach the border between the Central and Peripheral Zones from the center of the table and the total distance traveled were determined for each hamster on two consecutive days.

### Statistical methods for non-microbiome studies

All data were analyzed and graphed using GraphPad Prism v7 except for Dunnett’s test, which was performed with SPSS v22. A p-value of < 0.05 was considered significant. All graphs are generated using GraphPad Prism v7. For Fig. 1C,D, pairwise comparisons between infected and uninfected at each time point were made using Multiple T Test corrected for multiple comparisons using Holm-Sidak. For Fig. 2B,C (to test whether each group showed a preference to either object, *i*.*e*., preference score ≠ 0.5), each group was analyzed using a one-sample t-test comparing each of the means to 0.5 (two-tailed). For Figs 2B,D, 3B,D, 4B,C when comparing two groups to each other, a two-tailed Mann-Whitney test was used as the data were non-normally distributed. For Figs 2C and 3C, one-way ANOVA with one-tailed Dunnett’s post-test was used comparing each of the three infection groups to the uninfected group.

### Microbiome studies

Samples were derived from two independent experiments (A and B) as follows. For both experiments, the fecal collection was done inside the biosafety hood. During fecal collection, hamsters were individually placed in empty autoclaved cages supplemented with one layer of clean, dry paper towels on the bottom of the cages. Two to three fecal pellets were collected using sterile forceps right after voiding and immediately frozen on dry ice in sterile 15 mL conical tubes. All the fecal materials were kept at −80 until they were further processed. Experiment A included stool freshly collected and flash frozen from hamsters on consecutive days 32 and 33 post-infection from a total of 19 animals, 9 of which were infected. Experiment B included stool freshly collected and frozen on day 32 post-infection from 17 animals, 7 of which were infected. Of these 16 infected animals in total, ten could be classified as low infection burden and six classified as moderate infection burden based on fecal egg counts. DNA was extracted using Mo Bio PowerSoil DNA isolation kits following manufacturer’s instructions. 16S rRNA gene sequencing was performed by the DNA Sequencing and Genotyping Facility Core at Cincinnati Children’s Hospital Medical Center using the 319F and 806R primers as described^48^. Reads were generated as 300nt paired-end reads on the illumina MiSeq platform. Sample read data were demultiplexed using FASTX-Toolkit (http://hannonlab.cshl.edu/fastx_toolkit/index.html). Read data was processed to generate OTU tables with the UPARSE pipeline^49^ and classified using sintax (usearch v10.0.240_i86linux64). For all statistical analyses, sequences from both fecal collections were treated as a single data set. Alpha diversity was evaluated at a sampling depth of 14,000 reads per sample using Faith’s Phylogenetic Diversity (PD) index^50^. Taxa differentially associated with infection were determined using the Kruskal-Wallis test as implemented in QIIME (v1.9)^51^ and ANCOM^52^ as implemented in QIIME2 v2018.4. Beta diversity analysis was conducted using the unweighted UniFrac metric and significance assessed by pairwise PERMANOVA as implemented in QIIME2 v2018.4. Sequence data is deposited under NCBI BioProjectID PRJNA535443.

### Ethical approval

All animal experiments were carried out under protocols approved by the University of California, San Diego and University Massachusetts Medical School Institutional Animal Care and Use Committees (IACUC protocols S09067 and A-2483-14 respectively). All housing and care of laboratory animals used in this study conform to the NIH Guide for the Care and Use of Laboratory Animals in Research and all requirements and all regulations issued by the USDA, including regulations implementing the Animal Welfare Act (P.L. 89–544) as amended.
